# Combined transplantation of hiPSC-NSC and hMSC ameliorated neuroinflammation and promoted neuroregeneration in acute spinal cord injury

**DOI:** 10.1186/s13287-024-03655-x

**Published:** 2024-03-05

**Authors:** Xiaofeng Du, Desheng Kong, Ruiyun Guo, Boxin Liu, Jingjing He, Jinyu Zhang, Asiamah Ernest Amponsah, Huixian Cui, Jun Ma

**Affiliations:** 1 Hebei Medical University-Galway University Stem Cell Research Center, Hebei Medical University, Shijiazhuang, 050017 Hebei Province China; 2 Hebei Research Center for Stem Cell Medical Translational Engineering, Shijiazhuang, 050017 Hebei Province China; 3 Hebei Technology Innovation Center for Stem Cell and Regenerative Medicine, Shijiazhuang, 050017 Hebei Province China; 4 Hebei International Joint Research Center for Stem Cell and Regenerative Medicine, Shijiazhuang, 050017 Hebei Province China; 5 Department of Biomedical Sciences, College of Health and Allied Sciences, University of Cape Coast, PMB UCC, Cape Coast, Ghana; 6 Human Anatomy Department, Hebei Medical University, Shijiazhuang, 050017 Hebei Province China

**Keywords:** Spinal cord injury, Induced pluripotent stem cell, Mesenchymal stem cell, Neural stem cell

## Abstract

**Background:**

Spinal cord injury (SCI) is a serious clinical condition that has pathological changes such as increased neuroinflammation and nerve tissue damage, which eventually manifests as fibrosis of the injured segment and the development of a spinal cord cavity leading to loss of function. Cell-based therapy, such as mesenchymal stem cells (MSCs) and neural stem cells (NSCs) are promising treatment strategies for spinal cord injury via immunological regulation and neural replacement respectively. However, therapeutic efficacy is rare reported on combined transplantation of MSC and NSC in acute mice spinal cord injury even the potential reinforcement might be foreseen. Therefore, this study was conducted to investigate the safety and efficacy of co-transplanting of MSC and NSC sheets into an SCI mice model on the locomotor function and pathological changes of injured spinal cord.

**Methods:**

To evaluate the therapeutic effects of combination cells, acute SCI mice model were established and combined transplantation of hiPSC-NSCs and hMSCs into the lesion site immediately after the injury. Basso mouse scale was used to perform the open-field tests of hind limb motor function at days post-operation (dpo) 1, 3, 5, and 7 after SCI and every week after surgery. Spinal cord and serum samples were collected at dpo 7, 14, and 28 to detect inflammatory and neurotrophic factors. Hematoxylin–eosin (H&E) staining, masson staining and transmission electron microscopy were used to evaluate the morphological changes, fibrosis area and ultrastructure of the spinal cord.

**Result:**

M&N transplantation reduced fibrosis formation and the inflammation level while promoting the secretion of nerve growth factor and brain-derived neurotrophic factor. We observed significant reduction in damaged tissue and cavity area, with dramatic improvement in the M&N group. Compared with the Con group, the M&N group exhibited significantly improved behaviors, particularly limb coordination.

**Conclusion:**

Combined transplantation of hiPSC-NSC and hMSC could significantly ameliorate neuroinflammation, promote neuroregeneration, and decrease spinal fibrosis degree in safe and effective pattern, which would be indicated as a novel potential cell treatment option.

**Graphical abstract:**

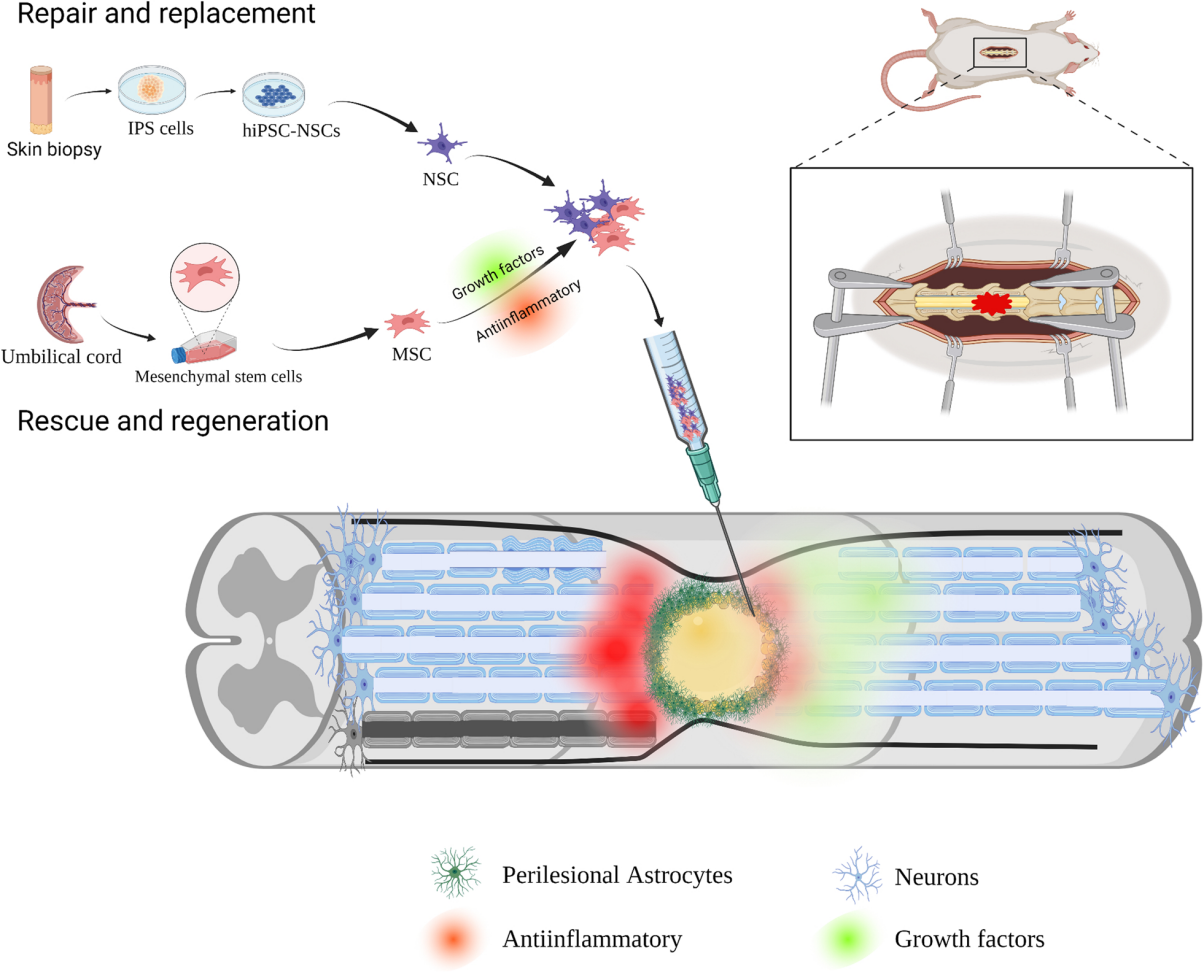

**Supplementary Information:**

The online version contains supplementary material available at 10.1186/s13287-024-03655-x.

## Introduction

Spinal cord injury (SCI) is a devastating clinical problem with severe neurological consequences, leading to long-term functional disability and paralysis [1]. According to the 2022 reports released from National Spinal Cord Injury Statistical Center, USA (NSCISC) and World Health Organization (WHO), there are 18,000 new cases of SCI every year with a total of 299,000 alive patients only in the USA and 500,000 new cases are added every year around the world thus incurring a huge management and financial burden on the healthcare system [2].

The pathological changes underlying acute SCI is a two-step process involving primary and secondary injuries and its ultimate severity depends both on the initial trauma, which physically destroys neurons or severs axons, and also on the extent of delayed secondary damage caused by inflammation, which leads to edema, neuronal apoptosis, cavitation, and reactive gliosis. During the secondary injury phase, neuronal death extends to adjacent segments, while neuroinflammation and the associated nonpermissive microenvironment limit the ability of adult neurons to spontaneously regenerate axons [3].

Current clinical management of SCI is limited to surgical intervention for spinal cord decompression and the administration of methylprednisolone, a corticosteroid with controversial efficacy [4]. These treatments rely heavily on the patient's limited capacity to self-repair and rebuild damaged nerve tissue. Stem cell therapy as an alternative strategy has great promise for the treatment of SCI [5], because stem cells are able to self-renew, differentiate into multiple lineages and secrete neurotrophic factors [6, 7] and neuroinflammation [8, 9]. Among several types of stem cells evaluated, neural stem cells (NSCs) are of particular interest, as they can replace lost host cells, rebuild neuronal circuitry and lost connectivity, provide nutritional support to promote functional recovery and play other roles in acute SCI [10]. The induced differentiation of human induced Pluripotent Stem Cell (iPSC) into NSC (hiPSC-NSC) provides a proliferative and broadly expandable in vitro cell source with glial and neuronal differentiation potential. More recently, we have reported that hiPSC-NSCs promote functional recovery in mice with acute SCI by replacing missing neurons and attenuating fibrosis and glial scar formation [11]. Meanwhile, the selection to use iPSCs and their derived cells does not present serious problems associated with genetic and epigenetic abnormalities and tumorigenic differentiation [12].

At the same time. Many related approaches have also conducted to explore the safety and efficacy of mesenchymal stem cells (MSCs) in the treatment of SCI [13–15], highlighting MSCs transplantation as a promising means to locomotor recovery in animal models [16]. Some recent studies indicated that MSCs are of favorable immunomodulatory, neuroprotective and neuroregenerative properties [17]. MSCs also display anti-apoptotic, angiogenic and lack tumorigenicity [18, 19]. Several studies have shown that when expanded with neurotrophic growth factors, MSCs can cross the germline and transdifferentiate into neuron-like cells [20–22]. In addition, MSCs can form a neuroprotective environment in the injured spinal cord by releasing neurotrophic factors such as nerve growth factor and neurotrophic factors and activating neurotrophic receptors thus effectively promoting the repair of spinal cord injuries [23, 24].In addition, MSCs can be easily harvested from the placenta because they are usually discarded by the parents at birth, thus effectively avoiding ethnic conflict.

Studies have shown that MSCs can regulate the growing microenvironment of NSCs and promote their survival rates [25]. At the same time, efforts are under way to further enhance the repair ability of NSCs through various means, including genetic modification and combining of NSCs into other cell types [25–27]. Therefore, this study was designed to observe the repair ability of combined treatment on SCI in mice by combining the immunomodulatory, neurotrophy effects of MSCs and the ability to provide a good living environment for NSCs.

## Materials and methods

### Ethics statement

All human sample collection and animal studies were approved by the Experimental Ethics Committee of Hebei Medical University (assurance no. 20190505). Title of an ethical approved project: Efficacy and mechanism of human iPSC derived neural stem cells in the treatment of spinal cord injury. Date of approval May 5, 2019. These experiments were performed in accordance with the National Institutes of Health Guidelines for the Care and Use of Laboratory Animals.

### Preparation of human umbilical cord mscs and human NSCs

#### Differentiation and identification of NSCs

Pluripotent Stem cell (PSC) neural induction medium (Gibco) induced iPSCs (cell line: HEBHMUi002-A, generated by our group) [28] to NSCs according to manufacturer's instructions. Simply put, iPSCs were cultured in PSC neural induction medium on a Geltrex-coated 6-well plate, with the medium changed every other day. iPSCs differentiated into NSCs, After 7 days [29]. The derived cells were fixed with 4% paraformaldehyde (Sigma, St. Louis, MO, USA) in DPBS (Invitrogen, Carlsbad, CA, USA) for immunofluorescence staining with the markers nestin and SOX2 [SRY (sex-determining region Y)-box 2] to identify the NSCs. Subsequently, NSCs were cultured in NSC amplification medium [30]. Passage 3 were used in our experiments.

#### MSC isolation and expansion

Briefly, the umbilical cord was cut into 3–4 cm long pieces under sterile conditions, placed on a petri dish containing saline solution and each section was cut along the umbilical vein, gently removing epithelial cells, venous, and arterial walls. The remaining tissue was cut with scissors into 1 mm^3^ pieces and fixed evenly at the bottom of the Petri dish. Complete MSC growth medium (Jingmeng, Beijing, China) was added to each Petri dish and placed in an incubator at 37 °C with 5% CO_2_. 14 days later, cell growth was observed. Further passages were made when the cells reached 90% confluence. Passage 4 were used in our experiments [11].

### Animals and surgical procedure

Eight-week-old immunodeficient BALB/c nude mice (*n* = 104, Beijing Vital River Laboratory Animal Technology Co., Ltd. Beijing, China), with the weight 19–22 g, were used for SCI model (eighty-four female mice were used to test the efficacy of cell transplantation and 10 female mice and 10 male mice were used to evaluate the safety of cell). All experimental procedures were approved by the Animal Ethics Committee of Hebei Medical University and followed the ARRIVE guidelines strictly. Animals were housed together under standard husbandry conditions with a 12-h light/dark cycle, with free access to food and water. Make every effort to minimize the number of animals used and their suffering. The surgical procedure was slightly modified according to previous report. The mice were anaesthetized using an overdose of ketamine–xylazine (Hubei XinRunde Chemical Co., Ltd, Wuhan, China). A laminectomy was performed at the 10th thoracic vertebra to expose the dorsal surface of the spinal dura mater. We elevated a weight of 10 g impactor (Zhongshi impactor, Beijing, China) to the fifth pore height, which is 5 cm, and fixed it with an iron plug. After that, the impactor was moved to the exposed surface of the spinal cord, the iron plug was withdrawn, and the impactor was released to establish a contusion SCI model. After skin closure, mice were placed on a warm mat until they were fully awake.

### Experimental groups and cell transplantation

Eighty-four nude mice were randomly divided into cell-free (PBS) and cell groups (hMSCs + hiPSC-NSC) (42 mice in each group), which are referred to as the Con and M&N group, respectively. Immediately after the SCI, 1 μl of PBS was injected into the injured spinal cord (1 mm rostral to the lesion epiCenter) [31] of 42 mice using a glass pipette at a rate of 0.5 μl/minute with a 10μl Hamilton syringe and a stereotaxic microinjector (RWD, Shenzhen, China). Using the same method, 42 mice were injected with 1 × 10^5^ cells/μl (0.5 × 10^5^ hMSCs and 0.5 × 10^5^ iPSC-derived NSCs) in M&N group. All surgeries were conducted under anesthesia, and efforts were made to minimize the animals suffering, with selected humane endpoints.

### Behavioral assessment

Functional recovery after SCI was assessed with the Basso Mouse Scale (BMS) scores [32]. Six mice from each treatment group were randomly selected for behavioral testing at days post-operation (dpo) 1, 3, 5, 7, 14, 21, and 28. All animals were placed on a flat surface with dimensions of 20 cm × 40 cm and observed for 3 min by two examiners in the randomized double-blinded pattern to the identity of the animals to evaluate the animals’ behaviors, including coordination, trunk instability, and stepping. A score of 0 represents flaccid paralysis; a score of 9 represents normal gait. For the stepping score, 0 indicates no stepping, and scores of 1, 2, and 3 indicating occasional, frequent and consistent dorsal stepping with no plantar stepping, respectively. Scores of 4, 5, and 6 represent occasional, frequent and consistent plantar stepping, respectively.

### Safety evaluation of transplanted cells

The selected experimental mice were fed adaptively for 14 days before administration. During the experiment, the mice were randomly divided into two groups according to gender and body weight: solvent control group and human iPSC-derived neural stem cells combined with human mesenchymal stem cells group (M&N group), 10 animals in each group, half male and half female, kept in cages at the same time. The control group was given PBS solution, while the M&N group was given 0.5 × 10^5^ hiPSC-NSC and 0.5 × 10^5^ hMSC. The model of administration is the same as that of modeling. After administration, the animals were closely observed for at least 2 h. Daily clinical observation was carried out from the second day after administration, and the observation was recorded for 16 consecutive days, in which the animals were weighed every other day after administration. At the end of the 16-days observation period, all the surviving animals were anesthetized using an overdose of ketamine–xylazine, then sacrificed and gross anatomy and gross observation of major organs were made.

### Measurement of serum cytokine levels

Serum cytokine levels were measured to evaluate the effects of two kinds of combined stem cells transplants (hMSCs and hiPSC-NSC) on inflammation at 7, 14, and 28 days after SCI (*n* = 6 animals per group at each time point). The blood was collected, incubated overnight at 4 °C and centrifuged at 5000 rpm, then serum was collected for ELISA to measure cytokines. According to the manufacturer's instructions, the mouse ELISA kits (Abclonal, Wuhan, China) were used for ELISA of Vascular endothelial growth factor (VEGF), Interleukin-6 (IL-6), Tumor necrosis factor-α (TNF-α), and brain-derived neurotrophic factor (BDNF). Nerve growth factor (NGF) level was analyzed using commercial ELISA kits (RayBiotech, American). The OD value at 450 nm (reference at 570 nm) was detected with SPARK (Tecan Trading AG, Switzerland), and calculate the absolute concentration from the standard curve.

### Histology and immunofluorescence staining

Six mice were randomly selected from each group and sacrificed at the time points of dpo 7, 14 and 28. Mice were subjected to terminal anesthesia with ketamine (80 mg/kg) and xylazine (40 mg/kg), perfusion with saline, followed by 4% paraformaldehyde (PFA) in PBS for fixation. The spinal cords were removed from the body, kept overnight in 4% PFA (4°C) and then cryoprotected overnight in 30% sucrose in PBS. The next day, the spinal cords were trimmed to the injured part (5 mm of total length), embedded in the TissueTek compound (Wetzlar, Germany), frozen and maintained at − 80 °C. Tissue sections of 14 μm thickness were obtained for hematoxylin–eosin staining (H&E) and Masson trichromatic staining.

### Western blot analysis

Mice were deeply anesthetized with ketamine–xylazine, and then spinal cord tissue (approximately 5 mm) was extracted from the epicenter of injury at indicated times (*n* = 6 animals per group at each time point). For sample processing, the tissues were homogenized in RIPA buffer supplemented 1 × protease inhibitor cocktail, sonicated and centrifuged at 12,000rpm for 20 min then the tissues were left on ice to lysis for 30 min. Protein concentrations were determined by the Pierce BCA method. Protein samples were run on 4–20% SDS-PAGE, and transferred to 0.45 μm nitrocellulose membrane. After blocking with 5% skimmed milk for 2 h, the membranes were incubated overnight with primary antibodies against GAPDH (mouse, 1:1000, Servicebio) and TGF-β (rabbit, 1:1000, Abcam) at 4 °C (Table 1). The membranes were washed with Tris-buffered saline containing 0.1% Tween20 (TBST), and then incubated with HRP-conjugated secondary antibody at room temperature for 2 h. The protein bands were detected by Image Quant Ai600 (General Electric Co., USA) with enhanced chemiluminescence reagent. The results were analyzed and quantified using ImageJ software (version 2.0.0, USA).

**Table 1:** Antibody information chart

Name of antibody	Company	Mono/polyclonal	Molecular weight (Kda)	Source	Dilution for WB	Dilution for IHC
GAPDH	Servicebio	Mono	36KD	Mouse	1:1,000	NA
TGF-β	Abcam	Mono	48KD	Rabbit	1:1,1000	NA

### Transmission electron microscope analysis

M ice were deeply anesthetized using ketamine–xylazine, then intracardiac perfused with saline, and then perfused with pre-cooled 2.5% glutaraldehyde fixative solution until the mouse were stiff (*n* = 6 animals per group at dpo 28). A sharp blade was used to quickly remove the tissue blocks of 0.5–0.7cm on ice which were obtained from the proximal and distal gray matter areas 2 mm from the center of the injured spinal cord, and then immersed in 4% glutaraldehyde for overnight fixation. Then the tissue was fixed with a 1% oxidizing fixative for 1 h, stained with 1% uranyl acetate for 2 h, and dehydration in a gradient acetone solution. Next, it was embedded in an oven at a temperature gradient at 45 °C for 24 h and 60 °C for 48 h. After semi-thin sectioning and toluidine blue staining, 60 nm tissue slices were cut with an ultrathin microtome (Leica UC7), stained with 1% uranium acetate-lead citrate, and finally observed the spinal cord structure under the TEM (model: Hitachi H-7500; voltage: 80 kV). For ultrastructural analysis, synaptic density, PSD thickness, length of synaptic activity zone and synaptic cleft width were calculated using the methods previously studied. TEM microphotographs of 10 fields of each section were taken in a simple random pattern to calculate synaptic density. A total of 120 synapses were analyzed to determine the PSD thickness, length of synaptic activity zone and synaptic cleft width, 20 synapses were examined for each mouse. The axon G-ratio was calculated by dividing the diameter of an axon by the diameter of axon plus the associated myelin sheath. Approximately 100–150 axons were used in 6 animals each group. The parameters were measured using Image J (version 1.50i, National Institute of Health, USA). And the results were compared by t test using Graphpad Prism. Statistical significance was set at *p* < 0.05.

### Quantitative RT-qPCR

Mouse spinal cord tissue (approximately 5 mm) was extracted from the epicenter at indicated times after injury (*n* = 6 animals per group at each time point). Subsequently, the supernatant was extracted by homogenization lysis and reverse transcription into cDNA using the First Strand cDNA Synthesis Kit (Supersmart™ 6 min 1st Strand cDNA Synthesis Kit). The volume of the whole reaction system was 20 μL. Gapdh was adopted as an internal reference gene for relative quantification. Calculation of the fold change of target mRNA expression relative to GAPDH based on the threshold cycle (CT) as *r* = 2^−Δ(Δ*CT*)^, where Δ*CT* = *CT* (target)-*CT* (GAPDH) and Δ (Δ*CT*) = Δ*CT* (experimental)-Δ*CT* (control). The sequences of primers used for RT-qPCR are listed in the Table 2 [33].

**Table 2:** Sequences of primers used for RT-qPCR

Gene	Forward primer sequence	Reverse primer sequence
BDNF	GGGTCACAGCGGCAGATAAA	GCCTTTGGATACCGGGACTT
NGF-β	GTTTTGCCAAGGACGCAGCTTTC	GTTCTGCCTGTACGCCGATCAA
VEGF	TCTTCAAGCCGTCCTGTGTG	CTCCAGGGCTTCATCGTTACA
TGF-β	ACTGGAGTTGTACGGCAGTG	GGGGCTGATCCCGTTGATTT
IL-6	GACAAAGCCAGAGTCCTTCAGA	TGTGACTCCAGCTTATCTCTTGG
TNF-α	GATCGGTCCCCAAAGGGATG	CCACTTGGTGGTTTGTGAGTG

### Quantitative analysis

The contours of the lesion area were manually drawn from longitudinal section images of the injury center and the area was calculated using Image J (version 1.50i, National Institute of Health, USA)[34]. Six high-power (× 200) Masson trichromatic staining fields were selected in each section, and Image-Pro Plus 6.0 software (Media Cybernetics, Silver Spring, MD, USA) was used to calculate the ratio of the spinal fibrosis area to the whole area [35]. For the inflammatory cell (such as neutrophile granulocyte) count, six digital photographs of 400 × 400 pixel square HE stained specimens was used to take near the center and counted inflammatory cells manually using Image J (version 1.50i, National Institute of Health, USA) [36].

### Statistical analysis

Statistical analyzes were performed using SPSS 22.0 (StatSoft, Tulsa, OK, USA) or GraphPad PRISM 9.0 (GraphPad Software, San Diego, USA) statistical software. All quantitative data are presented as the means ± standard errors of the means (SEM). Statistical analysis of multiple-group comparisons was performed by one-way analysis of variance (ANOVA), while the t-test was used for comparison between the two groups. Values of P less than 0.05 were considered statistically significant.

## Result

### Safety assessment of transplanted cells

**Fig. 1:**
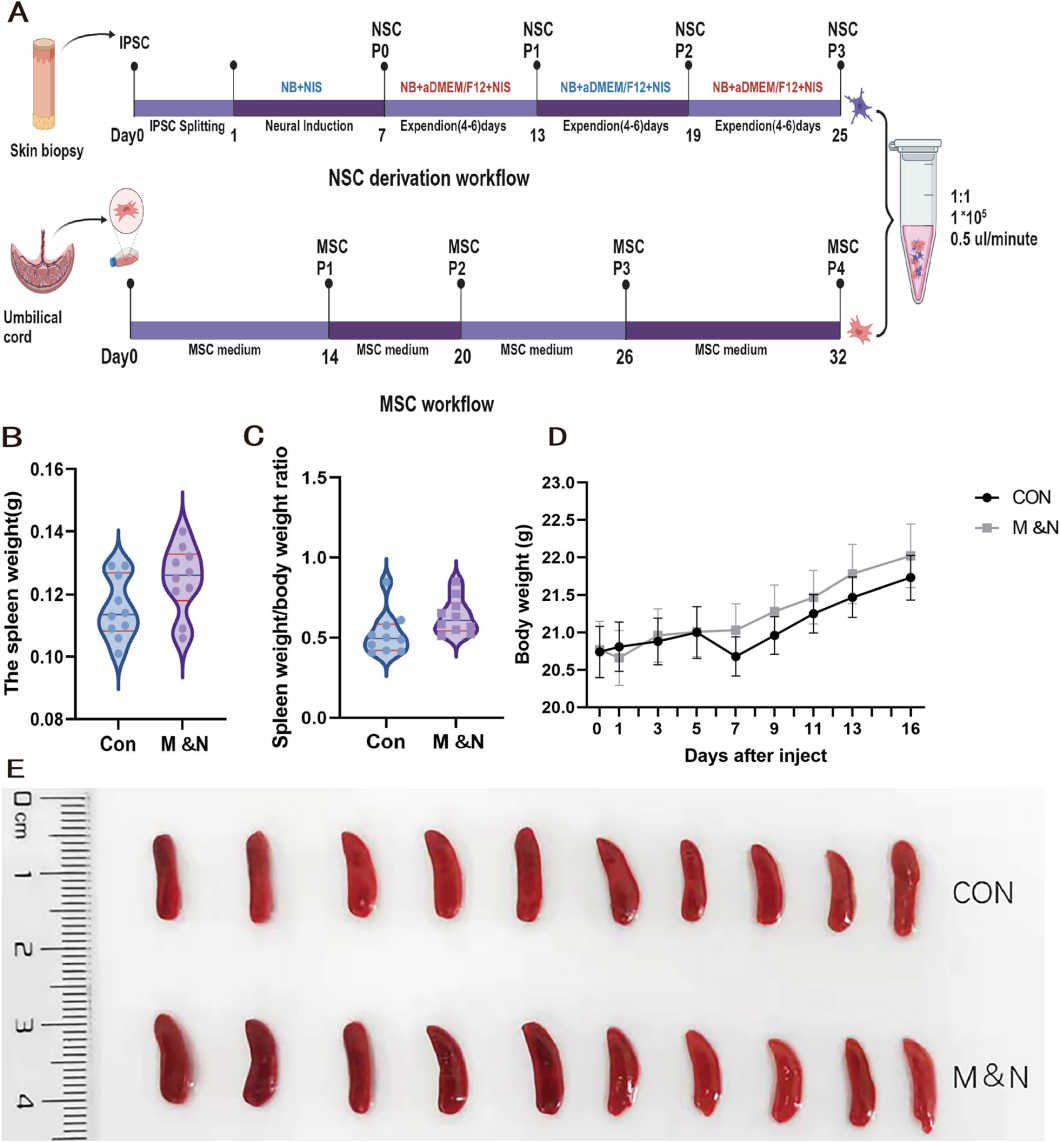
Cell acquisition and safety assessment after cell transplantation: iPSCs were cultured in PSC neural induction medium on a Geltrex-coated 6-well plate for 7 days, and iPSCs differentiated into NSCs. After 13 days, the cells were passaged as the first NSC generation, and the cells were cultured for another four to six days to obtain the second passage NSC. The third passage NSC was selected for the experiment. Human umbilical cords were cut and uniformly fixed at the bottom of the Petri dish, and cultured in an incubator at 37 °C with 5% CO_2_ after adding MSC medium. The passage was observed after 14 days, and the fourth passage was selected for the experiment. The final number of MSC and NSC cells was mixed 1:1 before treatment (**A**). **B** Spleen weights of mice were recorded after sacrifice. **C** Spleen weight-body weight ratio of mice was recorded after sacrifice. **D** Recordings of body weight weighing performed every other day after cell transplantation. **E** Size plot of mouse spleens after sacrifice

In order to evaluate the safety of transplanted cells in mice and whether they can differentially cause acute toxic reactions, we performed a safety evaluation of the cells according to the technical guidelines for toxicity studies of single drug administration released by the National Medical Products Administration of the People’s Republic of China ([2015] -46) for the single-dose toxicity studies of drugs. Mice were weighed at 1 day intervals after cell transplantation and the animal weights fluctuated slightly (Fig. 1D), but there were no significant differences between the groups (Table 3). On day 16, the animals were dissected and the spleens of all animals were weighed and counted. The overall spleen weight and organ ratio coefficients were greater in the NSC&MSC group compared to the lysate control group, but no statistical differences were seen (Table 4). No significant toxic changes were observed in the major organs of the animals by general observation (Fig. 1B–E). Therefore, our application of hMSCs + hiPSC-NSC was safe.

**Table 3:** Body weight of nude mice at different times after administration (g)

Group	Number	0 day	1 day	3 days	5 days	7 days	9 days	11 days	13 days	16 days
CON	F01	19.9	19.8	19.9	20.3	20.9	21.0	21.4	21.6	21.5
	F02	20.7	20.8	21.0	22.8	20.6	20.9	21.0	21.4	21.8
	F03	20.2	20.2	20.3	20.0	19.5	20.0	20.6	20.8	21.1
	F04	22.0	21.8	21.6	21.0	20.4	20.8	21.0	21.4	21.8
	F05	20.9	21.0	21.4	21.2	21.1	21.7	22.3	22.7	23.2
	F06	20.6	20.6	20.7	20.8	20.7	20.7	20.8	20.8	20.7
	F07	18.9	19.2	19.5	19.6	19.8	19.7	19.9	19.9	20.1
	F08	20.4	20.5	20.4	20.4	20.6	21.0	21.3	21.6	22.0
	F09	21.0	21.3	21.0	21.0	20.6	21.3	21.5	21.8	22.3
	F10	22.8	22.9	23.0	22.9	22.6	22.5	22.7	22.7	22.8
M & N	F11	19.8	20.0	20.6	20.3	20.1	20.6	21.0	21.5	21.8
	F12	19.7	19.8	20.0	20.2	19.8	19.7	20.0	20.4	20.7
	F13	21.6	21.8	22.0	21.9	21.8	22.0	21.9	22.2	22.3
	F14	22.1	22.0	21.8	21.5	21.7	22.0	22.6	23.8	24.3
	F15	19.1	19.1	19.4	19.5	19.4	20.0	19.9	20.4	20.7
	F16	20.0	20.4	20.7	21.0	21.2	21.3	21.4	21.6	21.6
	F17	21.8	21.5	20.9	20.5	20.5	20.6	20.6	20.5	20.8
	F18	21.9	20.0	22.2	22.2	22.0	22.4	22.3	22.3	22.6
	F19	22.0	22.4	22.5	22.8	23.0	23.2	23.4	23.7	24.2
	F20	19.7	19.6	19.5	20.2	20.8	21.0	21.6	21.4	21.2

**Table 4:** Spleen weight and spleen visceral-body ratio of nude mice

Group	Number	The spleen weight (g)	Spleen visceral-body ratio (%)
CON	F01	0.096	0.447
	F02	0.088	0.404
	F03	0.129	0.611
	F04	0.109	0.500
	F05	0.106	0.457
	F06	0.101	0.488
	F07	0.094	0.577
	F08	0.129	0.517
	F09	0.110	0.522
	F10	0.103	0.614
M&N	F11	0.152	0.697
	F12	0.165	0.771
	F13	0.141	0.632
	F14	0.109	0.449
	F15	0.206	0.972
	F16	0.135	0.654
	F17	0.127	0.879
	F18	0.140	0.523
	F19	0.130	0.450
	F20	1.122	0.585

### Efficacy evaluation of transplanted cells

#### Combined cell transplantation therapy promotes functional recovery after SCI in mice

**Fig. 2:**
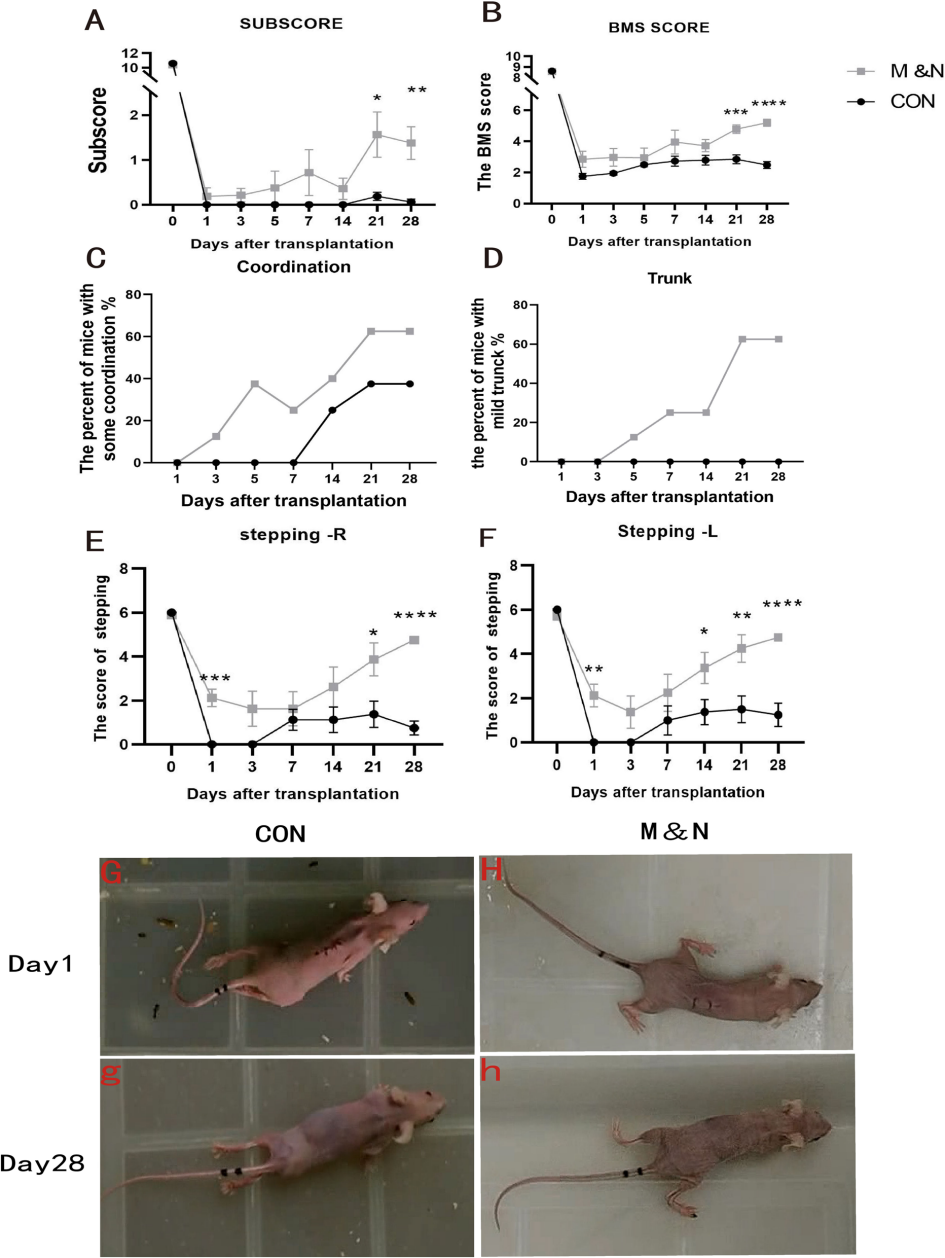
Recovery of motor function after stem cell transplantation in SCI. Assessment of motor function in PBS or stem cell treated mice using the BMS test: Subscore (**A**), BMS score (**B**), coordination (**C**), trunk (**D**), and the stepping score of right side (**E**) and left side (**F**). All animals in the M&N group showed significantly higher performance in the open-field BMS test than Con group (**B**). Significantly higher subscores were recorded for the M&N group than the Con group (**A**). With regard to coordination (**C**), in which stepping with body weight support is essential, a steady but insignificant trend toward higher performance was observed in the M&N group. Trunk function recovered earlier and to a greater extent in the M&N group than in the Con group (**D**). After stem cell transplantation, the M&N group showed a stable but non-significant trend of higher performance on stepping function at the early stage and from the dpo 7 onwards,showed a prominent increase (**E**, **F**). Mice limb function at dpo 1 (**G**, **H**); Mice limb function at dpo 28 (**g**, **h**). *p < 0.05; **p < 0.01

To investigate whether cell transplantation could lead to better functional recovery in mice with SCI, we first used the BMS motor function score to evaluate motor function in mice. At dpo 1, the hind limbs of mice in both groups were almost completely paralyzed (Fig. 2G, H, Additional file 1: Video S1, Additional file 2: Video S2). The difference in BMS score between the two groups was not statistically significant. The motor function of the mice in the M&N group gradually improved throughout the observation period, and decreased on dpo 14 compared with dpo 7. In contrast, motor function in the Con group mice recovered until dpo 21 and then decreased. At dpo 21 and 28, the M&N group scored significantly higher than the Con group (Fig. 2A). Interestingly, the animals transplanted in the M&N group all showed better BMS test scores for a shorter period of time after SCI (Fig. 2A, B). At dpo 28, the lower limb and hip muscles of control mice were significantly atrophied, while the muscle morphology of mice in the M&N group was close to normal (Fig. 2g, h, Additional file 3: Video S3, Additional file 4: Video S4). The score of transplanted animals in the M&N group reached a maximum, which was significantly higher than that of the Con group.

We also scored plantar gait speed, coordination, paw position, trunk and tail. At dpo 1, the scores of Con groups of animals decreased to zero. At dpo 28, the neurological function score in the M&N group was 1.5, which was significantly higher than the 0 score in the Con group (Fig. 2A). For changes in coordination, at dpo 28, 62.5% of the M&N group showed partial or substantial recovery of coordination compared to 37.5% of the Con group (Fig. 2C). Importantly, 62.5% of the M&N group showed mild trunk dysfunction compared to 0% of the Con group (Fig. 2D). Gait scores were significantly higher in both the left and right side of the M&N group than in the Con group, especially at dpo 21 and 28 (Fig. 2E, F). Thus, the combination cell therapy was very beneficial for the recovery of systemic functions (e.g., coordination and trunk function) in SCI mice.

#### Combined cell transplantation significantly reduces fibrosis levels after SCI

**Fig. 3:**
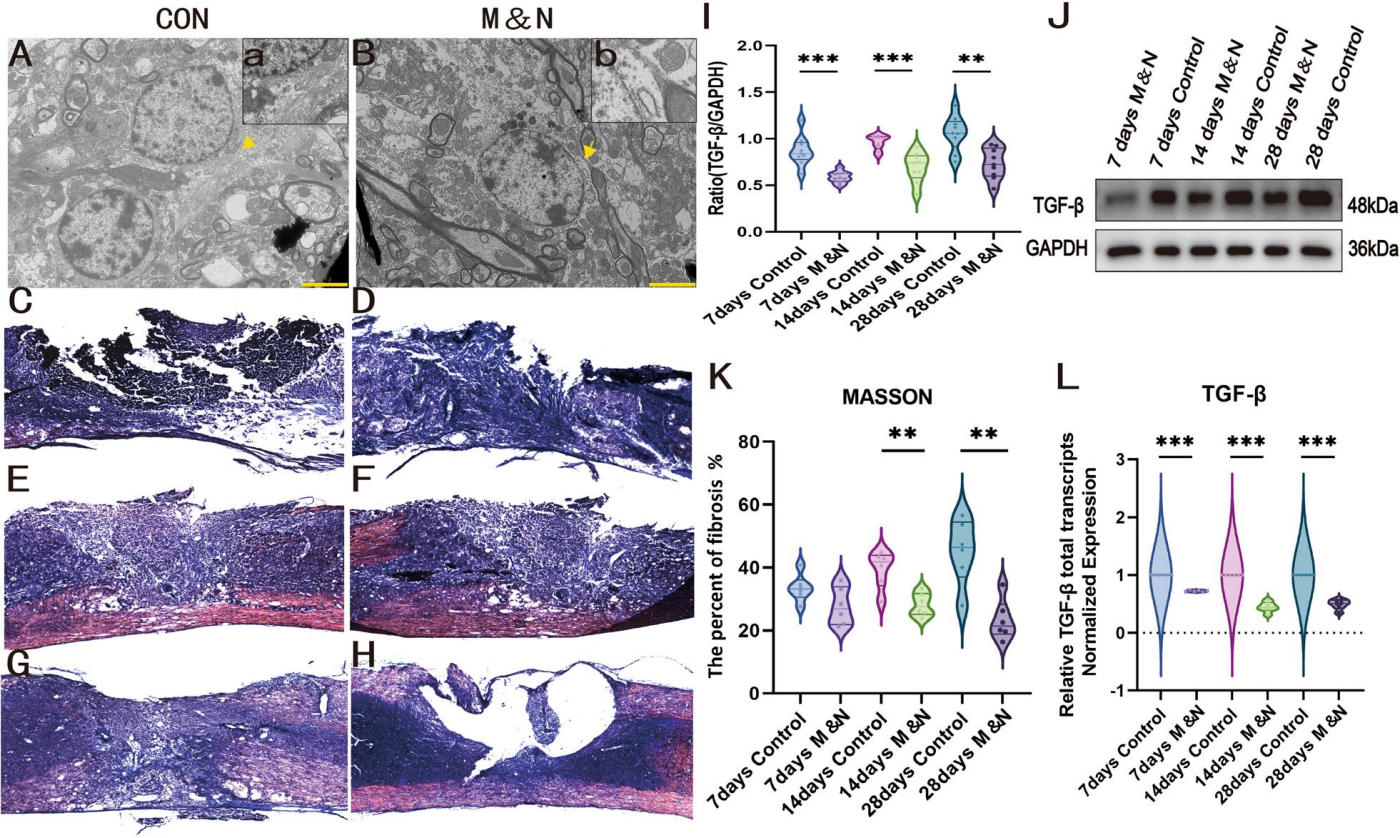
Cell transplantation can reduce the level of fibrosis after SCI. TEM showed A large number of a large number of intermediate filaments between the proliferating astrocytes in the Con group (**A**, **a**). The relative reduction in intermediate filaments between astrocytes after cell treatment (**B**, **b**). Scale bar, 5μm. Images of Masson’s trichrome staining in the different groups at dpo7 (**C**, **D**), dpo14 (**E**, **F**) and dpo 28 (**G**, **H**). The proportion of fibrosis in the M&N group was significantly lower compared to the Con group (**K**). Scale bar, 100μm. *p < 0.05; **p < 0.01; ***p < 0.001. The mRNA and protein levels of TGF-β were assessed by RT-qPCR and Western blot analysis (**L**, **J**). Western blot showed that the expression level of TGF-β in each group increased gradually with time (**J**) (Full-length blots/gels are presented in Additional file 5: Fig. S1). The expression of TGF-β was significantly decreased after cell transplantation in all three time periods compared with the Con group. The quantification of TGF-β expression (**I**). The data are expressed as the mean ± SEM; * p < 0.05; ** p < 0.01; ***p < 0.001

To observe whether cell transplantation improves fibrosis after SCI, the following experiments were performed. Under transmission electron microscope (TEM), we found that a large number of intermediate filaments appeared between astrocytes in Con group, while a relative decrease in intermediate filaments was observed under M&N group at dpo 28 (Fig. 3A, a, B, b). Meanwhile, TGF-β is a central pathological mediator of fibrotic disease, and it can respond well to the degree of disease fibrosis to some extent. The expression level of TGF-β after SCI was analyzed by western blotting (WB) and TGF-β showed an increasing trend at three incremental time points as the mice themselves recovered, while the TGF-β level in the M&N group at each time point was significantly lower than the Con group, which showed that cell transplantation reduced the level of fibrosis in SCI (Fig. 3I, J). Meanwhile, the mRNA level of TGF-β was consistent with the results of WB (Fig. 3L). To assess site injury scar formation, we calculated the percentage of fibrosis at the injury site by Masson staining. The area of fibrosis observed in the M&N group was smaller than in the Con group (Fig. 3C–H). The mean percentage of fibrosis in the M&N group was significantly lower than that in the Con group at dpo 14 and 28 (Fig. 3K). Taken together, these results suggest that combined cell transplantation significantly reduced the level of fibrosis in spinal cord injured tissues, thereby reducing the formation of scar tissue.

#### Cell transplantation affects inflammatory microenvironment after SCI

**Fig. 4:**
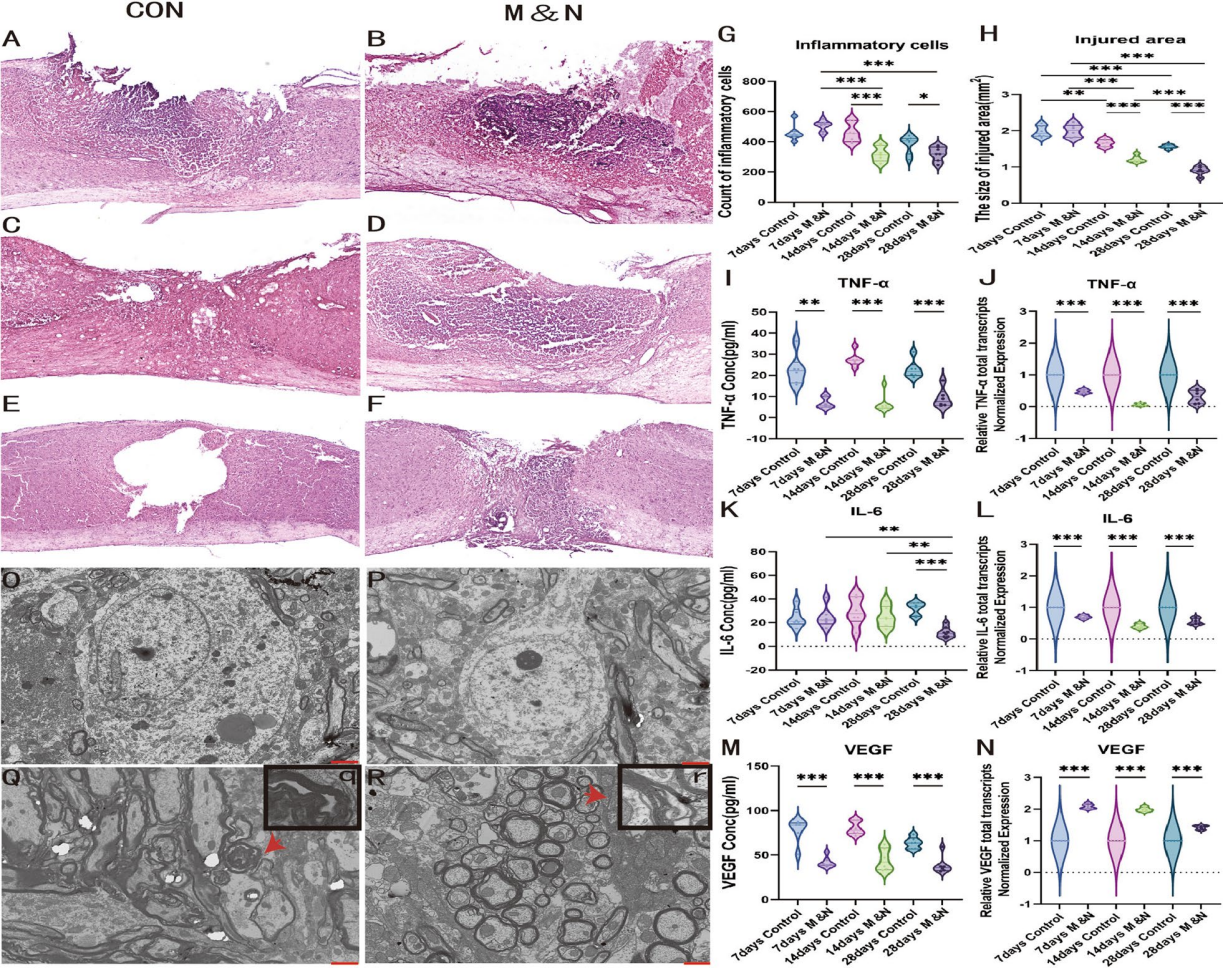
Cell transplantation suppressed the inflammatory response following SCI. Images of HE staining in the different groups at dpo 7 (**A**, **B**), dpo 14 (**C**, **D**) and dpo 28 (**E**, **F**). M&N group appeared a prominent lower level of inflammatory cell infiltration compared to the Con group at dpo 14 (**G**). The injured are size in Con and M&N group at dpo 28 and dpo 14 were significantly decreased compared to those at dpo 7, and the sizes in M&N group was significantly lower than that in the Con group at dpo 14 and dpo 28 (**H**). Scale bar, 100μm. *p < 0.05; **p < 0.01; ***p < 0.001. At the same time, the neuronal edema in the M&N group (**O**) was also alleviated compared with the Con group (**P**) under TEM. Demyelination was pronounced in the Con group (**Q**, **q**) compared with the M&N group (**R**, **r**). Scale bar, 5μm. The mRNA levels of IL-6, TNF-a, and VEGF in spinal cord tissue were assessed by RT-qPCR, and their serum levels were measured using ELISA analysis (**I**–**N**). The data are presented as the means ± SEM; *p < 0.05; **p < 0.01; ***p < 0.001

To test whether the neuroinflammatory response is attenuated after combined cell transplantation, we first performed HE staining, which revealed a large infiltration of inflammatory cells at the SCI site in the Con group, accompanied by morphological changes in spinal cord tissue. At dpo 14 and 28, the number of inflammatory cells in M&N group was significantly less than that in Con group. (Fig. 4A–G). At dpo 14, the Con group mice showed significant atrophy at the injury site, and tiny cavities could be observed to have appeared at the injury site, and at dpo 28, the cavities could still be observed and became large, but there was tissue continuity at the transection site. In contrast, no obvious atrophy was observed in the M&N group of mice, while the new tissue around the injured tissue gradually increased. The SCI sites of M&N and Con group recovered with time, and at dpo 14 and 28, the SCI sites of M&N group spanned a significantly smaller than that of Con group, and at dpo 28, the level of M&N was lower than that of other groups. (Fig. 4H). Subsequently, we performed cytokine assays. Elisa results showed TNF-a was lower in the M&N group than in the Con group at all three time periods, which was consistent with the results of RT-qPCR (Fig. 4I, J). Meanwhile, the results showed no difference in IL-6 levels between the two groups at dpo 7 and 14 and at dpo 28, IL-6 levels were significantly lower in the M&N group compared to dpo 14 and were lower than other levels at the same time point (Fig. 4K). Interestingly, there was no difference in IL-6 at dpo 7 and 14 in the ELISA results assay, however, in the RT-qPCR results, IL-6 levels were significantly lower in the M&N group than in the Con group (Fig. 4L). Compared with Con group, serum VEGF in M&N group was lower at all three time points (Fig. 4M). However, VEGF levels in the spinal cord mRNA expression were higher in the M&N group than in the Con group (Fig. 4N). Finally, we observed by TEM that the degree of neuronal edema in mice in the M&N group at dpo 28 was relieved compared to the Con group (Fig. 4O, P). At the same time, demyelination of the injured spinal cord was also alleviated (Fig. 4Q, q, R, r). This indicates that the neuronal damage was restored after cell therapy. In conclusion, the combined cell transplantation inhibited the release of inflammatory factors, reduced the level of inflammation in the SCI and improve the inflammatory microenvironment around the injured tissue, while alleviating the damage to the nerve cells after the injury.

#### Cell transplantation improves neuroplasticity after SCI

**Fig. 5:**
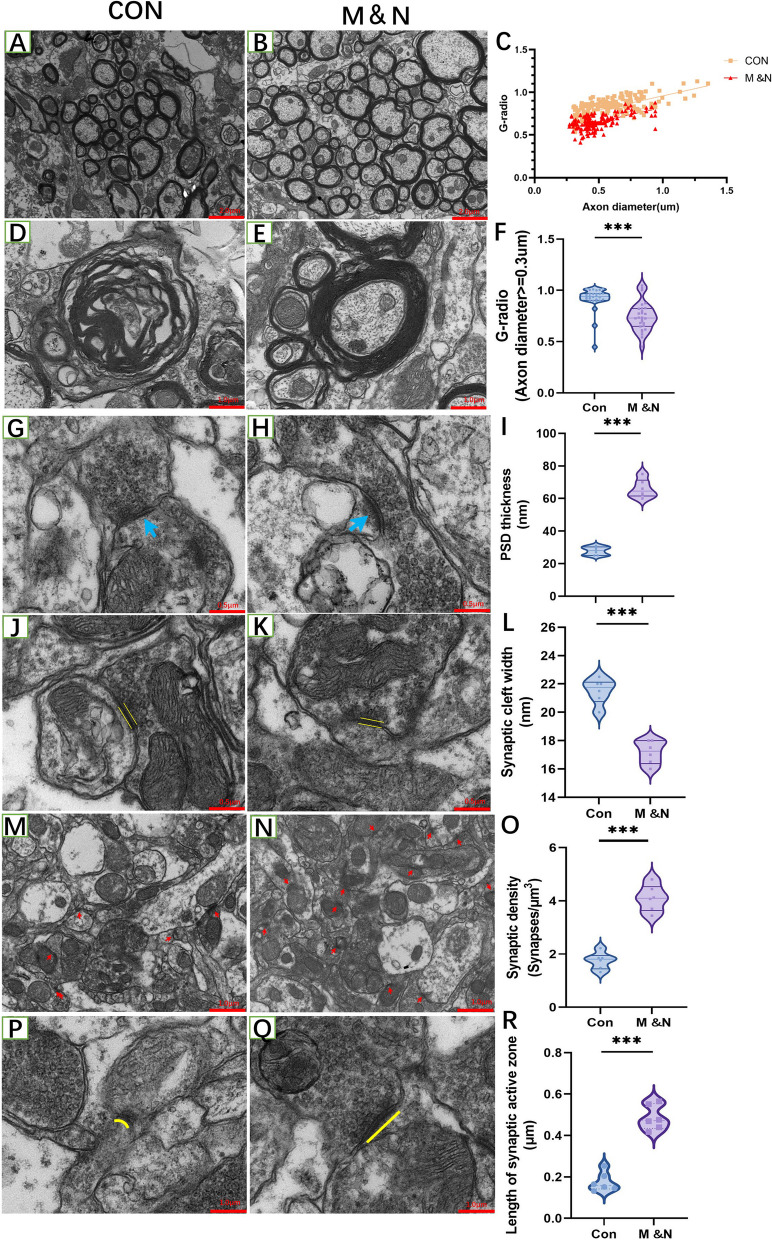
Cell treatment increased the repair conduction function after SCI. Demyelination was found to be alleviated in the M&N group by TEM (**A**, **B**, **D**, **E**), while the myelin G-radio values were smaller than those of the Con group (**C**, **F**), indicating that the cell treatment group had a thick myelin layer. TEM revealed significantly more postsynaptic dense material, number of synapses and postsynaptic active zone in the M&N group (**H**, **N**, **O**) than in the Con group (**G**, **M**, **P**). It was also found that the synaptic cleft in the M&N group (**K**) was smaller than that in the Con group (**J**). The data are presented as the means ± SEM; *p < 0.05; **p < 0.01; ***p < 0.001

Under TEM, the number of synapses, synaptic active zone and postsynaptic dense material in the M&N group were more than those in the Con group, while the synaptic cleft was narrowed in the M&N group (Fig. 5G–R). The myelin g-ratio is calculated as the ratio between the inner (*d*) and the outer (*D*) diameter of the myelin sheath: *g*-ratio = *d*/*D*. The smaller *g*-radio demonstrates the thicker myelin sheath layer. *G*-radio in the M&N group was lower than that in the Con group [37]. We also analyzed the relation between axon diameter and g-ratio (Fig. 5A–F).

#### Cell transplantation promotes the release of nerve growth-related factors

**Fig. 6:**
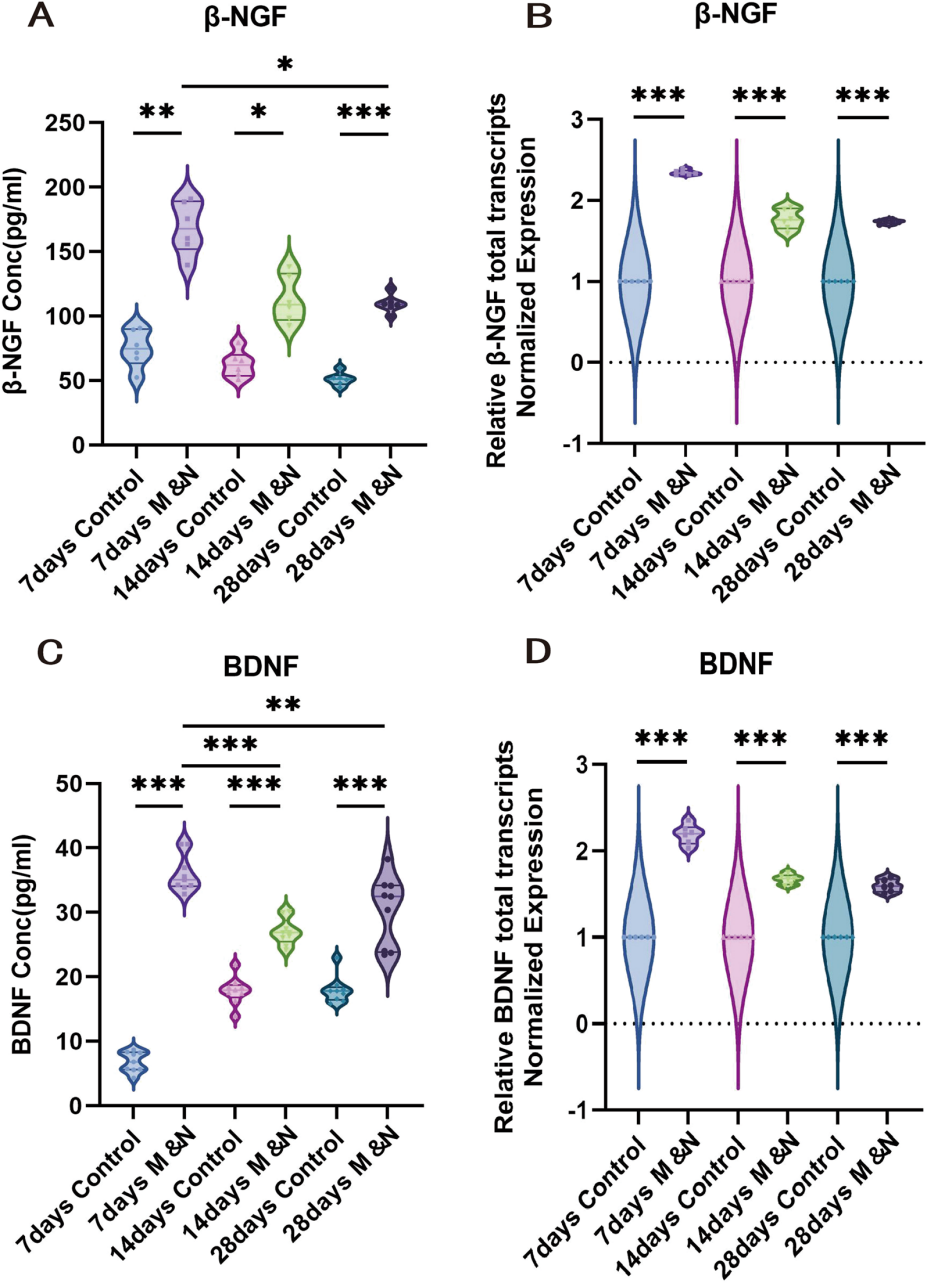
Cell transplantation can promote the release of nerve growth-related factors. The mRNA and serum levels of NGF (**A**, **B**) and BDNF (**C**, **D**) were assessed by RT-qPCR and ELISA analysis. The data are presented as the means ± SEM; *p < 0.05; **p < 0.01; ***p < 0.001

Our previous study confirmed that transplanted NSCs can survive and implant in the lesion area of SCI [11]. To determine whether the transplanted combined cells could maintain their advantage of secreting specific cytokines, we performed ELISA assays at dpo 7, 14 and 28 on the spinal cord as well as RT-qPCR assays. The results showed that NGF and brain-derived growth factor (BDNF) were significantly higher in mice after cell transplantation (Fig. 6A–D). In conclusion, combined cell transplantation enhanced the level of nerve growth-related factor.

## Discussion

Central nervous system injury inevitably leads to severe functional impairment. This is permanent neurological tissue damage caused by the inability of neurons to regenerate effectively. Currently, the available treatments remain largely unsatisfactory. Thanks to advances in stem cell transplantation therapy, this situation is gradually improving and there is growing evidence that treatment of traumatic neurological injury is possible. Combination therapy with stem cells, including MSC and NSC, has been reported for the treatment of SCI [12, 25, 38, 39], and our previous experiments confirmed that transplantation of NSC in the acute phase resulted in significant functional recovery in mice with SCI [11]. Similar studies have also shonw that transplantation of NSC can express neuronal [40] and glial markers and restore function in mice with SCI [41–43]. A number of previous studies have shown that MSC can effectively promote the recovery of spinal cord injury [44] and improve the motor function of mice by reducing the inflammatory response in vivo [45], promoting angiogenesis [46] and nerve fiber regeneration [47], and releasing neurotrophic factors such as BDNF [16, 24]. Both NSC and MSC have shown great potential for the treatment of SCI alone. Although some studies have shown that NSC combined with MSC transplantation can promote the preservation of motoneurons and myelinated tracts in rats with SCI [38, 48], there are few studies on the combination of NSC and MSC in the treatment of acute SCI in mice. This study attempts to explore the safety and efficacy of the combination of NSC and MSC on acute SCI in mice. Before treatment, we performed acute toxicological assays on the two combined cells and demonstrated that the combined cell transplantation did not produce acute toxic reactions in mice, which proved the safety of the combined transplantation. We then studied the effectiveness of the combined cell transplantation for SCI.

It has been shown that SCI induces an increase in the expression of pro-inflammatory cytokines (TNF-α, IL-6 and IL-1β) [49, 50]. In the present study, both ELISA and RT-qPCR showed that the combined treatment reduced the levels of IL-6, IL-1 and TNF-a. Also, HE results showed that at dpo 28, the level of inflammatory cell infiltration was lower in the combined treatment group than in the Con group. It indicates that the level of inflammation was reduced in both spinal cord tissue and in vivo in mice by cell therapy.

In addition to the pathological damage directly caused by trauma, one of the most important pathological processes in the injured spinal cord is the chronic progressive demyelination of axons [51], which improved after cell therapy in this experiment, recovery in spinal cord injured mice. This is consistent with the results of the BMS. The results of our study showed a significant improvement in the M&N group on day 1 after combined stem cell transplantation, as reflected in video and the score of stepping. This finding is surprising at first sight but is in part consistent with previous study that reported a rapid response to treatment [31, 52]. We speculate that this may be related to the synergistic effect of NSC and MSC, and their interaction in vivo may have contributed to the rapid physiological response and functional recovery. For the mechanism of rapid functional recovery and the interaction between NSC and MSC, it is also one of the focuses of our subsequent experiments. The results of VEGF detected in serum were contrary to those detected in the injured tissue. This finding suggests that local cell transplantation may promote vascular growth at the site of injury, while systemic VEGF levels did not show changes consistent with the local outcomes. Such localized effects, coupled with the lack of systemic changes, imply a more controlled and predictable intervention. Since the cell transplantation primarily affects the local environment (the site of injury) without causing unpredictable systemic alterations, this may enhance the safety of the treatment. Furthermore, the insignificant changes in systemic VEGF levels might also indicate that the treatment does not trigger widespread angiogenesis, thereby reducing the risk of adverse side effects. Therefore, although these observations cannot be taken as conclusive evidence of safety, they at least initially suggest the potential safety of local cell transplantation. Of course, a comprehensive evaluation of its safety requires further studies, especially long-term studies regarding the safety and side effects of the treatment. This is also an area that we need to explore more deeply in the future.

Infiltrating inflammatory cells (including microglia, fibroblasts, and reactive astrocytes) form fibro glial scars that limit regeneration of diseased axons [53]. Scar forming reactive astrocytes are densely distributed along the lesion edge, forming barrier like structures that express intermediate filament proteins after inflammation [54]. The scar tissue not only exerts a protective effect by limiting the spread of inflammation and secondary injury to adjacent intact tissue, but also acts as an inhibitory barrier to axonal regeneration [3]. Our experiments revealed a large reduction of intermediate filament between astrocytes under TEM after cell treatment. Also, Masson staining results pointed to reduced fibrosis and reduced glial scarring after cell treatment. TGF-β, a central pathological mediator of fibrotic diseases, to some extent, it reflects the degree of fibrosis of the disease. WB results indicated reduced TGF-β expression compared to controls. TGF-β results were likewise consistent with RT-qPCR results. Together, our pathological results and fibrosis-related indicators showed a reduction in fibrosis around the injured tissue. In conclusion, we conclude that the reduction of fibrosis degree and thus the reduction of scar tissue formation after cell therapy has a positive effect on the functional repair of SCI.

BDNF and NGF are a neurotrophic factor and nerve growth factor, respectively, that promote the regeneration, plasticity, and re-myelination of neurons in the nervous system in pathological states caused by disease or trauma [55–58]. Studies have shown neuroprotective effects by upregulating BDNF [59] and NGF [60] in the injured spinal cord. NSC grafts exert a neuroprotective and regenerative effects by upregulating BDNF [61] and NGF[62] in injured spinal cords. ELISA results showed elevated levels of BDNF and NGF in mice, suggesting that BDNF and NGF may be one of the trophic factors that enhance functional recovery in mice with SCI. Through these effects, transplanted cells can also induce neuroplasticity in the injured spinal cord by improving demyelination, increasing the number of synapses, postsynaptic compactness, and increasing synaptic active zone [42, 63, 64]. At the same time, under TEM we found that the *G*-radio of the M&N group was lower than that of the Con group, and the smaller *G*-radio indicated that the thicker myelin sheath layer.It is important to note that an increase in G-radio means a decrease in myelin thickness, a condition known as hypomyelination. The increase of *G*-radio in various diseases is associated with demyelination [65, 66]. We suggest that the treatment with stem cells creates a microenvironment that promotes nerve repair and nerve growth by decreasing the inflammatory response in mice while increasing the expression level of nerve growth factors. At the same time, stem cells also reduce the level of fibrosis around the SCI, thereby reducing the formation of scar tissue. The combined efforts of multiple factors thus promote SCI repair in mice. This experiment demonstrates that combined treatment may be a new protocol for the treatment of acute phase SCI in mice.

## Conclusion

According to our results, combined transplantation of hiPSC-NSC and hMSC could significantly ameliorate neuroinflammation, promote the secretion of neurotrophic factors, decrease spinal fibrosis degree and jointly create a microenvironment suitable for repair and regeneration of injured nerves in safe and effective pattern. Therefore, hiPSC-NSC and hMSC transplantation for acute SCI promotes the recovery of limb function, suggesting that hiPSC-NSC and hMSC are a promising cell type for SCI treatment.

### Supplementary Information

**Additional file 1**. **Video S1**: The video of mouse in control group at dpo 1.

**Additional file 2**. **Video S2**: The video of mouse in M＆N group at dpo 1.

**Additional file 3**. **Video S3**: The video of mouse in control group at dpo 28.

**Additional file 4**. **Video S4**: The video of mouse in M＆N group at dpo 28.

**Additional file 5**. **Figure S1**: Uncropped figure of WB in this study.

## Data Availability

The data that support the findings of this study are available from the corresponding author upon reasonable request.

## Abbreviations

SCI: Spinal cord injury
NSCs: Neural stem cells
iPSC: Induced pluripotent stem cell
hiPSC-NSCs: Human iPSC-derived NSCs
MSCs: Mesenchymal stem cells
PSC: Pluripotent stem cell
BMS: Basso mouse scale
TEM: Transmission electron microscope
TGF-β: Transforming growth factor-β
WB: Western blotting
NGF: Nerve growth factor
BDNF: Brain-derived growth factor
HE: Haematoxylin-eosin
TNF-α: Tumor necrosis factor-α
IL-6: Interleukin-6
dpo: Days post-operation
VEGF: Vascular endothelial growth factor
