# Supplementary material for: Combined transplantation of hiPSC-NSC and hMSC ameliorated neuroinflammation and promoted neuroregeneration in acute spinal cord injury
Source: Stem Cell Res Ther. 2024 Mar 5;15:67. doi: 10.1186/s13287-024-03655-x (PMC10916262; doi:10.1186/s13287-024-03655-x)
Supplement: Supplementary file 5 — Additional file 5. Figure S1: Uncropped figure of WB in this study. [file 13287_2024_3655_MOESM5_ESM.pdf]

Supplementary Figure. Uncropped figure of WB in this study and the explanation of Fig. 3J

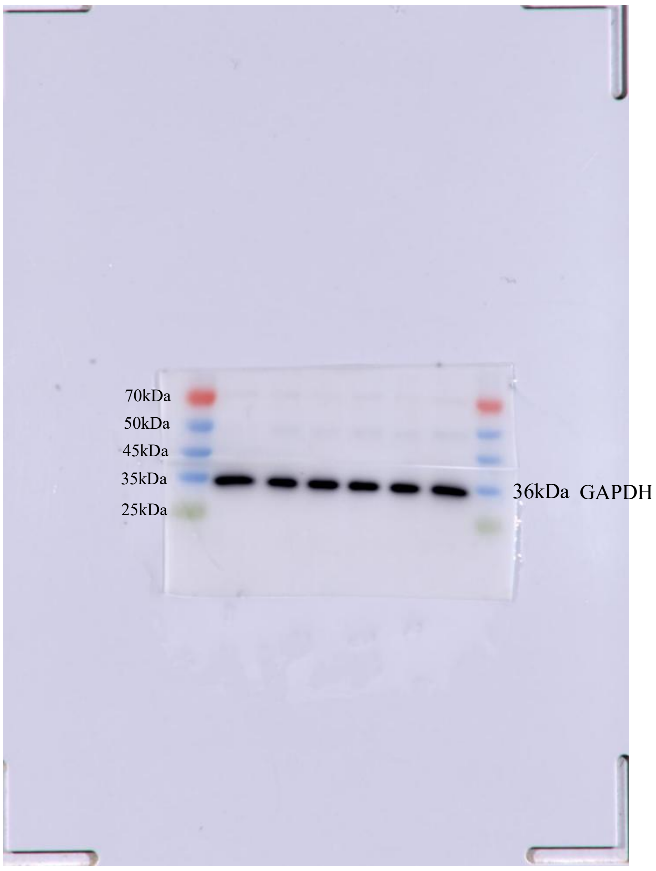

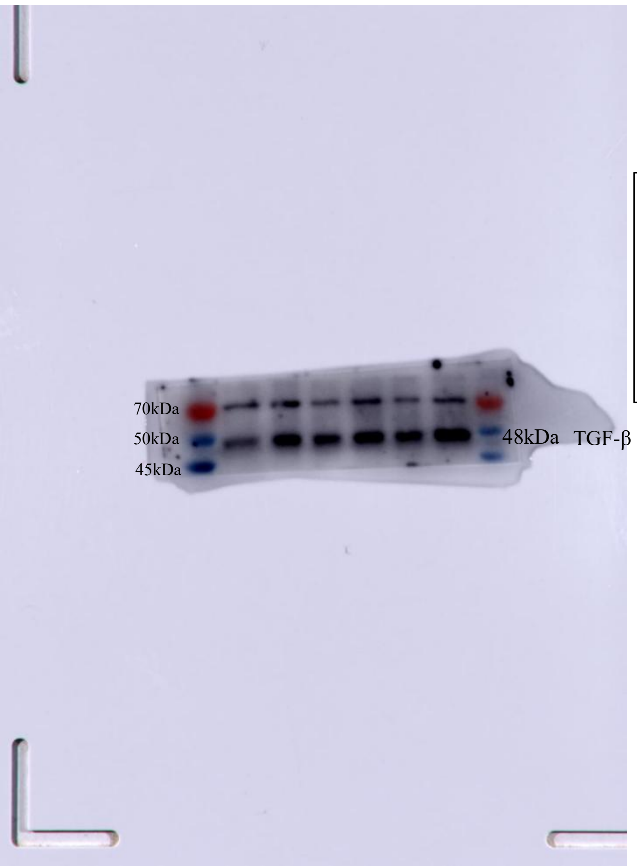

The band above the TGF- β is the trace of an experiment we did to validate the Transglutaminase 2 protein. In order to show the whole PVDF membrane, TG2 at the red mark is not cut out. There is no conflict between the two.
